# Draft Genome Sequence of *Acinetobacter johnsonii* C6, an Environmental Isolate Engaging in Interspecific Metabolic Interactions

**DOI:** 10.1128/genomeA.00155-17

**Published:** 2017-04-20

**Authors:** Rolf Sommer Kaas, Hanne Mordhorst, Pimlapas Leekitcharoenphon, Jacob Dyring Jensen, Janus A. J. Haagensen, Søren Molin, Sünje Johanna Pamp

**Affiliations:** a Research Group for Genomic Epidemiology, Technical University of Denmark, Kongens Lyngby, Denmark; b Novo Nordisk Center for Biosustainability, Technical University of Denmark, Kongens Lyngby, Denmark

## Abstract

*Acinetobacter johnsonii* C6 originates from creosote-polluted groundwater and performs ecological and evolutionary interactions with *Pseudomonas putida* in biofilms. The draft genome of *A. johnsonii* C6 is 3.7 Mbp and was shaped by mobile genetic elements. It reveals genes facilitating the biodegradation of aromatic hydrocarbons and resistance to antimicrobials and metals.

## GENOME ANNOUNCEMENT

*Acinetobacter johnsonii* C6 (formerly, *Acinetobacter* sp. strain C6) was isolated in 1994 from a microbial community of a creosote-contaminated aquifer at a gasworks in Fredensborg, Denmark (1, 2). Creosotes are mixtures of chemicals formed during natural gas production, which can contain aromatic hydrocarbons and a variety of heterocycles. Despite their toxicity, creosotes were used as medical treatment against infections, toothache, gastrointestinal, and respiratory complications.

*A. johnsonii* C6 forms biofilms and participates in interspecific interactions, including metabolic interactions, with *Pseudomonas putida* (3–6). The genetic determinants for these activities are largely unknown. Here, we report the draft genome sequence of *A. johnsonii* C6. It was generated using Illumina MiSeq sequencing (2 × 250 cycles), yielding 593,389 raw read pairs and a depth of coverage of ~68×. The reads were trimmed and filtered using bbduk2 (BBMap 35.82) (http://jgi.doe.gov/data-and-tools/bbtools/) and assembled using SPAdes 3.7.0 (7). Contigs smaller than 500 bp or with coverage below 2× were removed. The draft genome is 3,705,435 bp in 26 contigs, with a G+C content of 41.7%. It contains 3,543 genes, as predicted using Prodigal (8), 77 tRNA genes, and one rRNA operon (16S, 23S, 5S). The 16S rRNA gene sequence had >99% sequence similarity to *A. johnsonii* XBB1 (accession no. NZ_CP010350.1), *A. johnsonii* ATCC 17909^T^ (accession no. Z93440.1), and *A. johnsonii* DSM 6963 (accession no. X81663.1) (9–11). Putative functions for predicted proteins were assigned using PROKKA 1.1 and by comparing sequences to the public databases Pfam, KEGG, InterPro, and CARD (12–16), followed by submission-ready file conversion (https://bitbucket.org/RolfKaas/gff3_to_ena_embl).

*A. johnsonii* C6 encodes proteins predicted to convert aromatic hydrocarbons, such as benzyl alcohol, benzoate, fluorobenzoate, dihydroxybenzoate, methylcatechol, methylbenzyl alcohol, hydroxybenzaldehyde, hydroxymethylnaphthalene, naphthalenemethanol, benzene, toluene, chlorobenzene, and cyclohexanol. Previously, it was shown that this strain could grow on toluene, benzyl alcohol, and benzoate (4, 5).

A number of antimicrobials, as well as heavy metals (e.g., arsenate, mercury, tellurite, copper, and chromate), may be tolerated by *A. johnsonii* C6, mainly facilitated by proteins involved in their efflux, transport, reduction, and functions encoded by antibiotic resistance genes, such as *bla*_OXA-334_ (OXA-211 family) and *catB*. *In vitro* assays revealed that *A. johnsonii* C6 was resistant to chloramphenicol, trimethoprim, cefoxitin, and quinupristin-dalfopristin. *A. johnsonii* C6 may produce secondary metabolites, and it harbors biosynthetic gene clusters for a siderophore, aryl polyene, bacteriocin, and unknown metabolites, based on predictions by antiSMASH (17).

The *A. johnsonii* C6 draft genome encodes 19 proteins containing GGDEF and/or EAL domains involved in c-di-GMP metabolism, and proteins involved in motility (pili), and secretion (type II secretion system [T2SS], T6SS, secretory-signal recognition particle [Sec-SRP], and Tat), suggesting dynamic interactions with their environment, including with other microorganisms. The presence of features related to plasmids, phages, and insertion sequence (IS) elements suggests that mobile genetic elements have shaped the evolution and ecology of *A. johnsonii* C6.

The genome sequence of *A. johnsonii* C6 will facilitate the understanding of its physiology, evolution, and interaction with *P. putida*. Studies on *A. johnsonii* could also provide new insight into the biodegradation of aromatic hydrocarbons and resistance to antimicrobials and toxic metals, with relevance to environmental biotechnology.

### Accession number(s).

The draft genome sequence of *A. johnsonii* C6 is available from DDBJ/ENA/GenBank under the accession number FUUY00000000.
